# Feasibility and safety of breast-conserving surgery via a periareolar incision for cancers located far from the nipple–areolar complex: a retrospective study

**DOI:** 10.1007/s00432-020-03385-6

**Published:** 2020-11-04

**Authors:** Joohyun Woo, Jihae Lee, Se Hyun Paek, Woosung Lim

**Affiliations:** 1 Department of Surgery, Ewha Womans University College of Medicine, Ewha Womans University Mokdong Hospital, Seoul, Korea; 2 Department of Radiation Oncology, Ewha Womans University College of Medicine, Ewha Womans University Mokdong Hospital, Seoul, Korea; 3 Department of Surgery, Ewha Womans University College of Medicine, Ewha Womans University Seoul Hospital, Seoul, Korea

**Keywords:** Periareolar incision, Breast-conserving surgery, Breast cancer, Oncologic outcome, Cosmesis

## Abstract

**Purpose:**

We performed breast-conserving surgery (BCS) using periareolar incisions for cancers located far from the nipple–areolar complex (NAC) and examined if BCS via a periareolar incision maximized cosmesis and maintained oncologic safety. One of the most important goals of BCS is to improve cosmesis after surgery and quality of life, but the skin incision can affect cosmesis based on the tumor location.

**Methods:**

Fifty-five patients with breast cancers located far from the NAC underwent BCS via periareolar incisions between January 2017 and April 2018. If a sentinel lymph node biopsy was required, another skin incision was created in the axilla using the conventional technique. Medical records of patients were reviewed retrospectively.

**Results:**

The mean patient age was 48.1 ± 10.6 years. The mean tumor size was 1.8 ± 1.0 cm (range 0.2–4.5 cm) on preoperative magnetic resonance imaging (MRI); the mean distance from the NAC to the tumor was 5.9 ± 1.9 cm (range 4.0–12.3 cm). Patients with cancers in the subareolar area were excluded even though the distance from the nipple was > 4 cm on MRI. Negative microscopic margins were obtained in all patients. There was no surgical complication such as seroma, bleeding, or infection. Re-operation was not needed. All patients received whole breast radiation therapy. After surgery and radiation therapy, periareolar incision scars were nearly invisible.

**Conclusion:**

For cancers located far from the NAC, BCS via periareolar incisions is feasible and leads to superior cosmesis in selective patients. Moreover, BCS seems oncologically safe, although long-term outcomes need to be evaluated.

## Introduction

Breast-conserving surgery (BCS) is a standard surgical treatment for localized breast cancer. Long-term survival of BCS followed by radiation therapy is equivalent to that of mastectomy (Litière et al. 2012). One of the most important goals of BCS is to improve cosmetic results after surgery; also, cosmetic results affect quality of life even several years after treatment (Bromberg et al. 2018; Hau et al. 2013).

Various types of skin incisions can be used for lumpectomy: curvilinear, transverse, radial, periareolar, and inframammary incisions. As surgeons usually place an incision in the breast over or near the cancer, the skin incision is dependent on the cancer location (Zollinger et al. 2011). A curvilinear incision along the Langer’s lines or a transverse incision along the Kraissl’s lines, the natural skin lines of the breast, are usually used because they can be utilized irrespective of the location of the cancer in the breasts. However, the skin incision is important not considering the access to the cancer, but for cosmetic results. In most cases, a transverse incision directly over the cancer gives a good cosmetic result compared with a curvilinear incision, which is associated with distortion or asymmetry and always produces a scar. If a cancer is located near the axilla, lumpectomy and sentinel lymph node biopsy are possible through a single radial incision. However, it may result in a long suture line across normal skin creases, which leads to excess contraction and deformity (Sabel 2009). When an incision is made in the medial quadrant, especially near the sternum, it is easily observed as a thick and obtrusive scar. When the cancer is below the nipple, incisions in the lower half of the breast can result in misalignment of the nipple. Although a radial incision prevents this deformity, it is usually avoided considering a future mastectomy incision (Shrotria 2001). The locations of the skin incision used for hidden scar lumpectomy are in the skin crease of a patient’s arm fit, an inframammary fold that is a natural skin crease or around the nipple–areolar complex (NAC). Periareolar or inframammary incisions tend to give the best cosmetic result (Zollinger et al. 2011). Among them, a periareolar incision can make be used to approach the cancer irrespective of the quadrant it is located in. Owing to the limitations of the other incisions and the best results associated with a periareolar incision, we performed lumpectomy using a periareolar incision for cancers far from the NAC and retrospectively examined whether this procedure maximized cosmesis and maintained oncologic safety by evaluating the pathologic and cosmetic outcomes.

## Methods

### Patient selection

A total of 562 patients diagnosed with ductal carcinoma in situ or invasive breast cancer underwent BCS in Ewha Womans University Mokdong Hospital from all breast surgeons between January 2017 and April 2018. Removal of cancers located far from the NAC via periareolar incisions was performed by one breast surgeon. Of the 105 patients who received BCS from this breast surgeon, we analyzed retrospectively 55 patients who received lumpectomy via periareolar incisions although their cancers were located > 4 cm from the nipple.

This technique was performed in patients who have indications for BCS, although their cancers were located > 4 cm from the nipple. The distance between the cancer and the nipple was measured from the edge of the areola to the nearest tumor border on magnetic resonance imaging (MRI). Patients with multifocal cancers eligible for BCS were included. Patients with suspicious local infiltration adjacent to primary tumor, involving skin or subcutaneous fat layer by direct extension in physical exam or ultrasonography were excluded. Patients with cancers located in the subareolar area were excluded even if the distance from the nipple was > 4 cm on MRI.

### Surgical technique

**Fig. 1:**
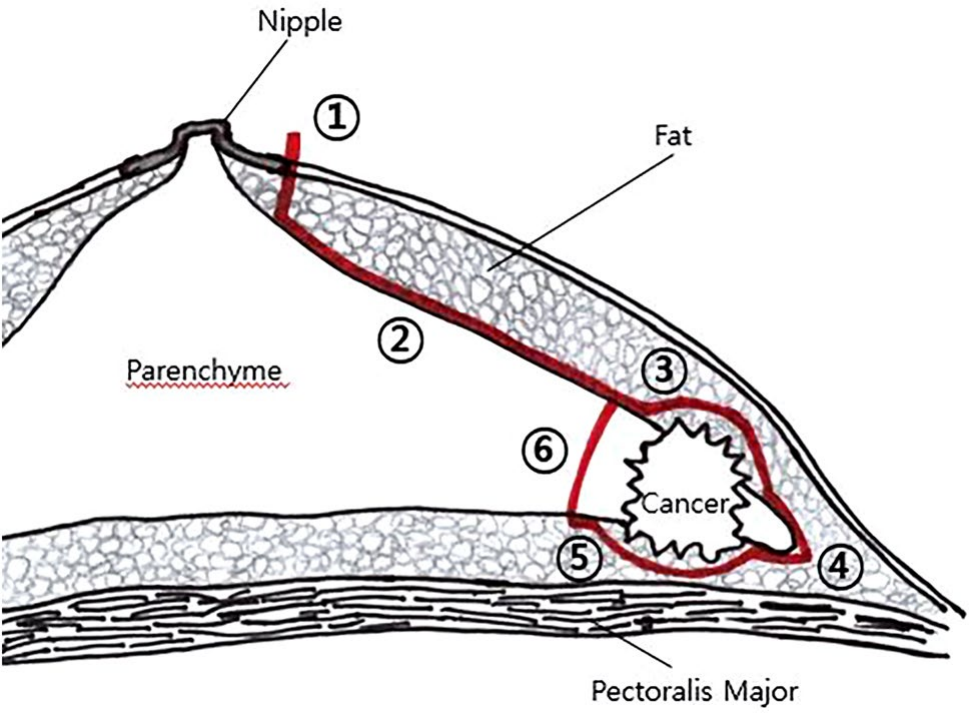
Schema of lumpectomy via a periareolar incision

The surgical procedure performed is shown in Fig. 1. The patient was placed in a supine position with the arm on the same side as the breast cancer abducted by 90°. The operation table was slightly tilted away from the main surgeon. Elevation of the arm was not necessary even if an axillary dissection was planned.

A skin incision was made sharply with a No. 15 scalpel blade—that has a small curved cutting edge—along the periareolar line along the depth of the epidermal layer. The length of the skin incision was determined to be adequate to allow good exposure and complete removal of the cancer, and it did not exceed half of the areolar circumference.

Once a periareolar incision was made using a scalpel, skin flaps were elevated with Allis clamps. Allis clamps were preferred, as they minimize skin damage compared to towel clamps. The dissection was performed vertically downward to the normal parenchyma until the normal breast parenchyma was exposed (① of Fig. 1). When the normal breast parenchyma was identified, subcutaneous dissection proceeded towards the cancer along the superficial surface of the breast parenchyma while saving all the adipose tissue (② of Fig. 1). The extent of dissection towards the cancer was determined by palpation with the non-dominant index finger, as it is best not to palpate the cancer directly using forceps or clamps. Presurgical localization was performed for patients with the tumor was not palpable on preoperative physical exam. Mammography or ultrasound-guided wire localization was performed on the day of surgery. When the cancer was detected with a small amount of covering fat, the dissection plane was tilted slightly upward from the breast parenchyma in order to not expose the cancer (③ of Fig. 1).

After dissection around the cancer, full exposure of the normal breast tissue around the cancer was attempted with fat preservation. Counter-nipple direction was divided firstly at the peripheral end of the breast parenchyma (④ of Fig. 1), and except direction to the nipple, the other 2 lateral breast parenchyma were excised with adequate margins. After the breast tissue was divided in three directions, the edge of the counter-nipple direction was elevated using a mosquito clamp, after which further dissection was attempted to access the plane between the parenchyma and retromammary fat tissue. In the same manner as superficial dissection, fat in the deep (posterior) area should be preserved except just beneath the cancer (⑤ of Fig. 1). If fat tissue just below the deep margin was adequately preserved, the pectoralis major was not visible. Lastly, the breast parenchyma from the margin to the nipple was divided because this approach from the periphery to the nipple allowed easy determination of adequate margins (⑥ of Fig. 1) and preserve the fat beneath the cancer. The order of direction in excision was important to minimize the amount of excised tissue and determine adequate margins. Preservation of superficial and posterior fat was important in maintaining the normal breast contour, but preservation of fat beneath the cancer (or above the pectoralis fascia) was technically very difficult.

**Fig. 2:**
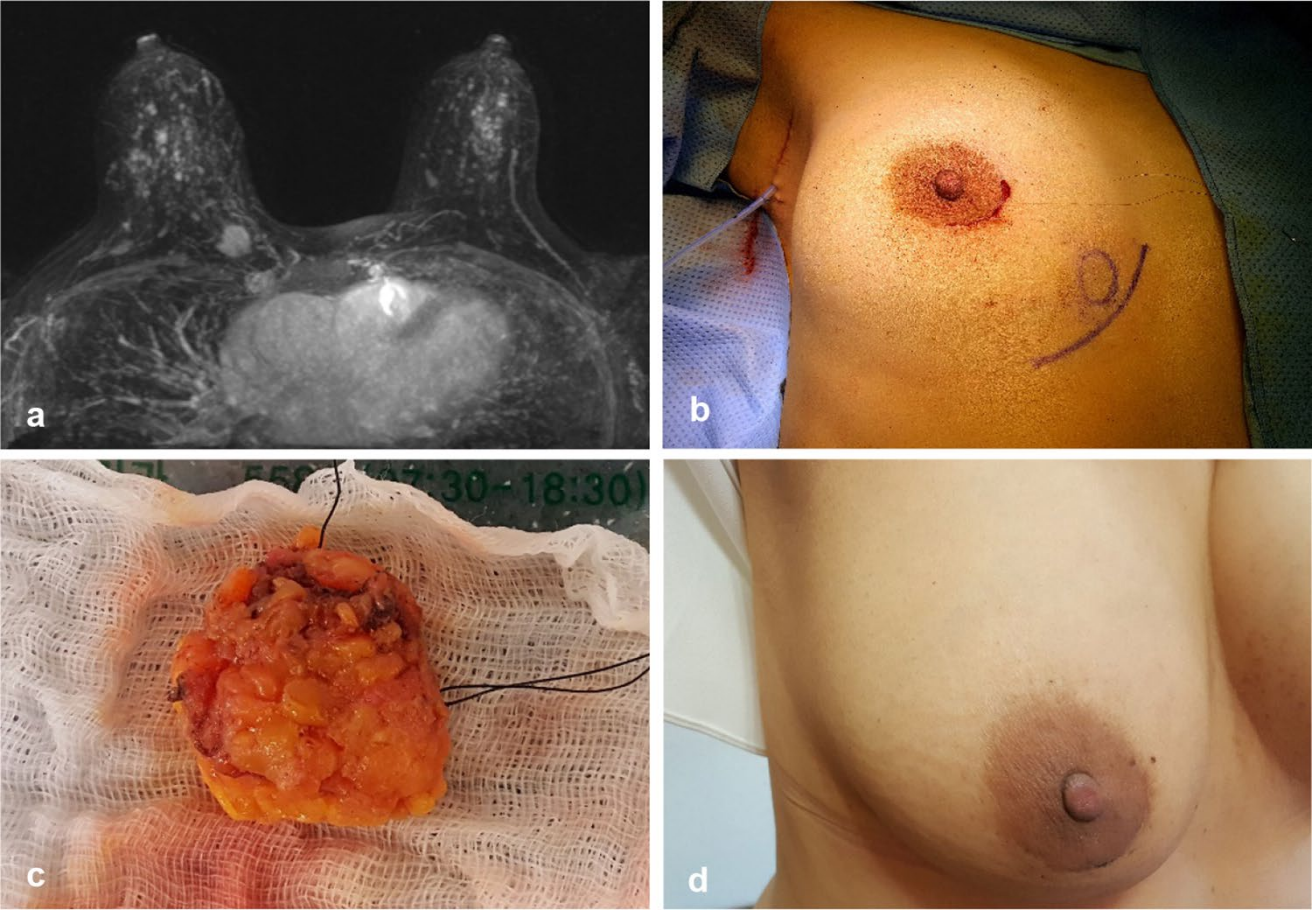
**a** A 44-year-old woman had breast cancer in the lower inner quadrant of the right breast, and the distance from the edge of the nipple–areolar complex to the cancer was 8.8 cm. **b** She underwent lumpectomy via a periareolar incision and sentinel node biopsy via a small axillary incision. **c** The largest diameter of the lumpectomy specimen was 2.7 cm, and negative surgical margins were observed on frozen section examination and pathology. She was diagnosed with T1 (1.5 cm) N0 and received 4 cycles of docetaxel and cyclophosphamide chemotherapy followed by radiation therapy. **d** After radiation therapy, the periareolar scar was nearly invisible

Because a small amount of superficial or deep fat covering the cancer was not related to local recurrence (Yoon et al. 2018), minimal amount of fat was included in the specimens where cancers were not exposed (Fig. 2c); all the preserved fat resulted in the best cosmetic result.

A drain was not necessary for simple lumpectomy, but if sentinel lymph node biopsy or axillary dissection was performed simultaneously along with lumpectomy, a closed drain (Hemovac^®^) was used to prevent seroma collection or hematoma formation.

**Fig. 3:**
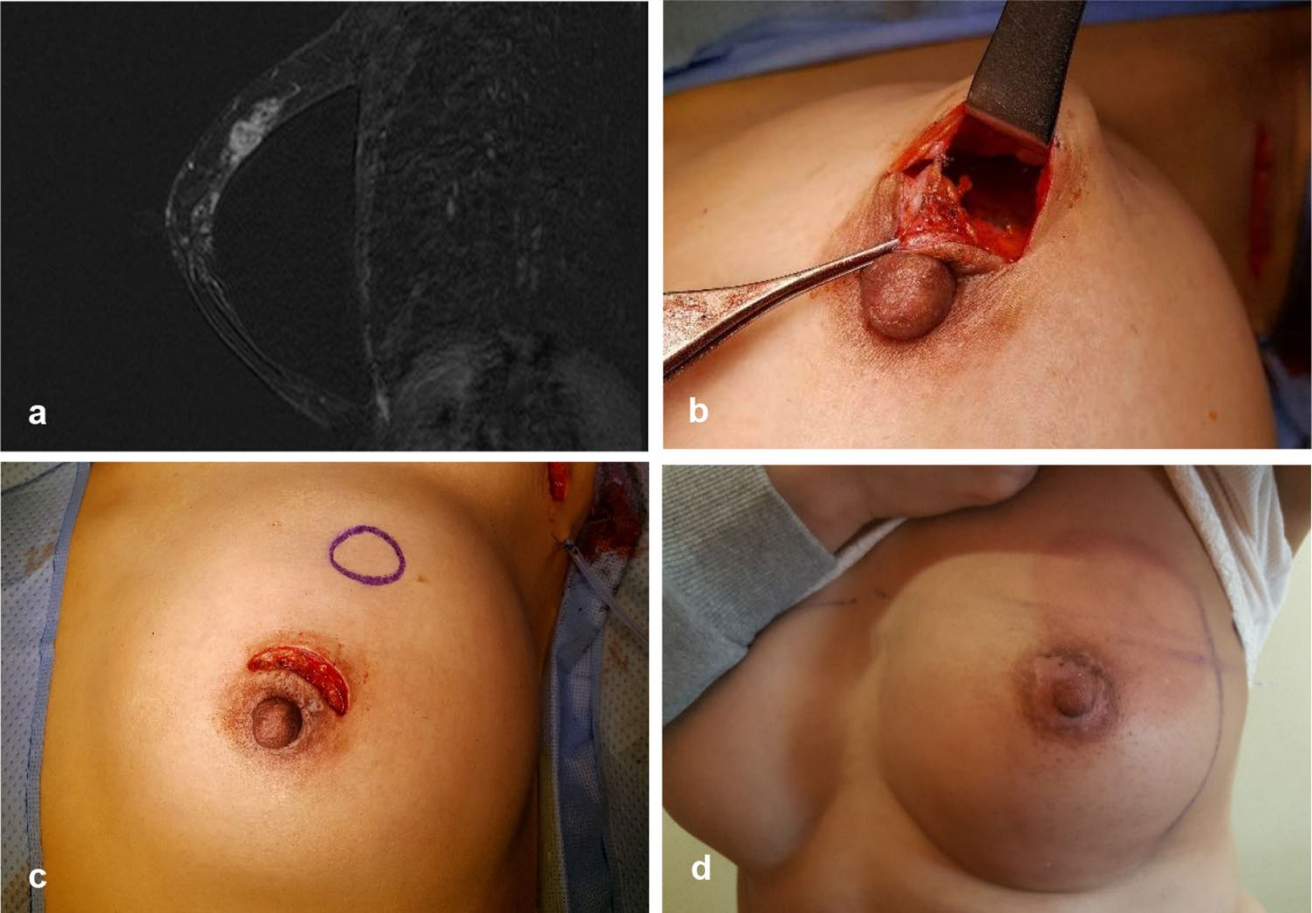
**a** A 2-cm cancer was located 4.3 cm from the nipple–areolar complex in a 48-year-old woman with augmentation implants. **b** Lumpectomy was performed successfully via a periareolar incision and axillary lymph node dissection. **c** After lumpectomy, the implant was saved and not exposed. **d** The patient received 4 cycles of doxorubicin and cyclophosphamide chemotherapy followed by weekly paclitaxel; after radiation therapy, the periareolar scar was nearly invisible and the contracture of the implant was minimal

**Fig. 4:**
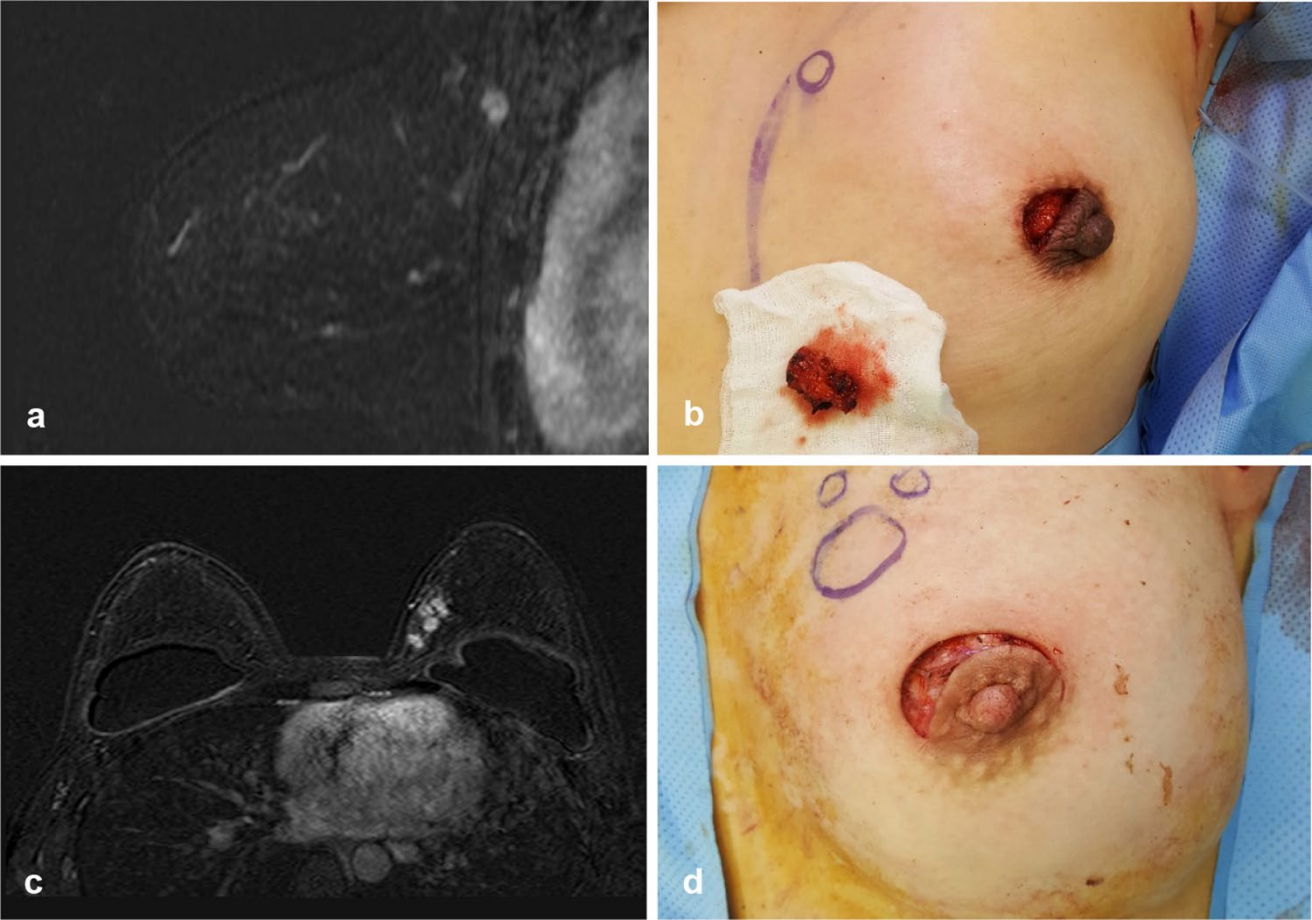
**a** A 48-year-old woman had a 1.4-cm cancer in the left upper inner quadrant, 12.3 cm from the edge of the nipple–areolar complex on magnetic resonance imaging (MRI). **b** Lumpectomy was performed through a periareolar incision. **c** Multifocal breast cancer was diagnosed in a 36-year-old woman with breast implants. The cancers were located in the upper inner quadrant of the left breast, 6.5 cm from the NAC on MRI. **d** The patient underwent lumpectomy via a periareolar incision and axillary dissection

Only one drain was used to cover both lumpectomy and axillary sites (Figs. 2, 3, 4). After lumpectomy was completed, long Kelly clamps were used for penetration from the lumpectomy cavity to the axillary area through the retromammary space. The end of the drain with pores was clamped by using long Kelly clamps and pulled from the axilla to lumpectomy the cavity. The other end of the drain was taken out through the skin below the axilla and an anchoring suture was placed using nylon sutures. The length of the drain was adjusted to place pores in both the lumpectomy cavity and the axilla.

This technique can be used safely even if the lumpectomy site is located across the nipple from the axillary incision. Because the retromammary space consists of loose connective tissue, penetration through this space by using long Kelly clamps is a safe procedure, with bleeding from this procedure occurring rarely.

The subcutaneous layer was closed with interrupted 3–0 coated polyglactin 910 with Triclosan (VICRYL Plus^®^ Antibacterial Suture) sutures. Interrupted subcuticular sutures of 4–0 polyglyconate (Maxon) were used for skin closure. The wound was covered using topical wound adhesives (Epibond^®^).

Postoperatively, no compressive dressing was applied in the breast and axilla. The drain was removed when the amount of drainage fluid for a day was less than 30 mL, after observation for 2 consecutive days.

Adjuvant treatment was performed according to standard guidelines. All the patients received radiation therapy. The clinical progress of patients within 6 months after BCS was considered for the evaluation of complications and cosmetic results.

This study was approved by the institutional review board of the Ewha Clinical Trial Center at Ewha Womans University Medical Center, and written informed consent was obtained from all the patients. Statistical analyses were performed using the IBM SPSS version 20 (SPSS^®^ Inc. Chicago).

## Results

A total of 55 patients underwent lumpectomy via the periareolar incision. The mean age of patients was 48.3 ± 9.6 years, and 32 patients were premenopausal.

Mean breast volume was 576.2 ± 317.3 mL (Table 1). Breast volume in the lower quartile were less than 330 mL and that in the upper quartile were above 830 mL. Breast volume was calculated using craniocaudal view of mammography because cup size can be affected by circumference of chest wall (Kalbhen et al. 1999; Ringberg et al. 2006). Regarding glandular density, more than two-thirds of the patients had heterogeneously dense breast on mammography (Table 1). It was classified into 4 groups according to breast imaging-reporting and data system (BI-RADS).

**Table 1:** Clinical characteristics of patients

	*N* (%) or mean ± SD (range)
Total patients	55 (100)
Age (years)	48.3 ± 9.6 (25–77)
Menstruation status	
Premenopausal	32 (58.2)
Postmenopausal	23 (41.8)
Breast volume (mL)	576.2 ± 317.3 (71.6–1227.7)
Glandular density	
Almost entirely fatty	1 (1.8)
Scattered areas of fibroglandular density	5 (9.1)
Heterogeneously dense	42 (76.4)
Extremely dense	7 (12.7)
Tumor size on MR^a^ (cm)	2.3 ± 1.1 (0.8–4.5)
Distance from NAC^b^ (cm)	5.9 ± 1.3 (4.0–12.3)
Tumor location	
Upper inner quadrant	12 (21.8)
Upper outer quadrant	28 (50.9)
Lower inner quadrant	5 (9.1)
Lower outer quadrant	10 (18.2)
Multifocality	3
Yes	4 (7.3)
No	51 (92.7)
Previous augmentation with implants	
Yes	3 (5.5)
No	52 (94.5)

^a^Magnetic resonance

^b^Nipple–areolar complex

The mean tumor size was 1.8 ± 1.0 cm on preoperative MRI, and the mean specimen size after lumpectomy was 4.8 ± 1.6 cm. The mean distance from the NAC to the tumor was 5.9 ± 1.9 cm. The diameter of the largest tumor was 4.5 cm, and the maximum distance from the NAC to the tumor was 12.3 cm (Tables 1, 2).

**Table 2:** Pathologic characteristics and surgical details

Variables	*N* (%) or mean ± SD (range)
Size of invasive component (cm)	1.8 ± 1.0
AJCC^a^ staging	
0	12 (21.8)
I	31 (56.3)
IIa	5 (9.1)
IIb	7 (12.7)
T stage	
Tis	12 (21.8)
T1	32 (58.2)
T2	11 (20.0)
N stage	
N0	47 (76.4)
N1	8 (14.5)
Hormone receptor	
Positive	47 (85.5)
Negative	8 (14.5)
HER2^b^ overexpression	
Yes	3 (5.5)
No	52 (94.5)
Preoperative wire localization	
Yes	7 (12.7)
No	45 (87.3)
Size of lumpectomy specimen	4.8 ± 1.6 (2.0–8.5)
Type of operation	
Lumpectomy only	11 (20.0)
Lumpectomy with SNB^c^ only	35 (63.6)
Lumpectomy with axillary dissection after SNB	9 (16.4)
Operation time (min)	
Lumpectomy only	51.9 ± 7.5
Lumpectomy with SNB only	66.6 ± 13.3
Lumpectomy with axillary dissection after SNB	98.6 ± 13.0

^a^The 7th edition of American Joint Committee on Cancer staging system

^b^Human epidermal growth factor receptor 2

^c^Sentinel lymph node biopsy

Negative margins were observed in intraoperative biopsy on frozen section analysis and permanent section evaluation. There were no complications such as seroma, bleeding, or infection. None of the patients required re-operation. Considering wound healing, there were no cases of necrosis of the skin edge, NAC ischemia, or nipple paresthesia. Lumpectomy via a periareolar incision was performed in 3 patients who underwent previous augmentation mammoplasty using implants; they received radiation therapy without complications after lumpectomy. All the patients were followed up at least once after surgery. The minimum follow-up duration was 1 month. During the follow-up period, cosmetic results were very good and the scar was almost invisible. There were no subjective complaints by the patients about areolar collapse and nipple paresthesia. Representative cases are shown in Figs. 2, 3, and 4.

## Discussion

This study is the first report on BCS using a periareolar incision for the breast cancer far from NAC. Although an approach using a periareolar incision is feasible for removal of breast masses, the efficacy has been evaluated in patients with benign breast tumors (Kong et al. 2016; Liu et al. 2011). On comparing fibroadenoma excision through a periareolar incision and through an overlying incision, the periareolar incision technique yielded better cosmetic results with minor postoperative complications. A significantly longer operation time or a larger volume of intraoperative blood loss is meaningless in the clinical field. Patients with an areola diameter > 3.5 cm, the distance between the fibroadenoma and the areola < 5.0 cm, and the largest diameter of the fibroadenoma < 3.0 cm were included (Liu et al. 2011).

The main difference between surgery for malignant and benign tumors is the margins. In breast cancer, it is very important to achieve complete excision with clear margins in order to reduce local failure and improve outcomes (Rahman 2011). The traditional incision for lumpectomy should ideally be made directly over the mass. In contrast, via a periareolar incision, a cancer far from the NAC can be resected by tunneling between the breast parenchyma and subcutaneous fat layer. Tunneling too far can be a concern when lumpectomy is performed using a periareolar incision. Excessive tunneling is not recommended because this may compromise the margins and make re-excision for positive margins unnecessarily difficult (Rahman 2011). However, the current study showed that a periareolar skin incision did not disturb the margins and helped in obtaining clear margins that maintain oncologic safety. There are risk factors related to a positive margin such as the extent of excision, age, large tumor size, multifocality, lobular histological type, and the number of positive lymph nodes (Yıldırım 2009). However, based on the results of this study, if the cancer with a diameter < 2 cm is removed to an adequate extent, the use of a periareolar incision does not threaten the oncologic safety. Nevertheless, we cannot suggest the cutoff value for the upper limit of the tumor size because of the small sample size.

On the basis of the principle in cancer surgery, it is important not to expose the cancer (Cady 1997). Physicians should be cautious when the cancer involves the surface of the breast parenchyma or reaches the subcutaneous tissue. In these cases, it is relatively easy not to expose the surface of the cancer when an overlying incision is made. In this study, lumpectomy through a periareolar incision was possible even when the cancer was not trapped within the breast parenchyma as long as the localization of the cancer was clear. If the cancer was abutting the subcutaneous or retromammary fat tissue, it could be removed with a thin layer of fat tissue. The minimum amount of subcutaneous fat over the cancer or retromammary fat below the cancer is excised so that the cancer is not exposed. However, even though superficial and deep margins are positive, it is not an important predictor for local recurrence (Yoon et al. 2018).

The better cosmetic outcome of a periareolar incision has been demonstrated (Liu et al. 2011; Saad and Kay 1984). A periareolar incision leaves no obvious scar and causes less severe breast deformity in most patients. Moreover, most patients do not complain about the breast scars owing to a periareolar incision. When the cancer is in the inner quadrant of the breast, especially near the sternum, using a periareolar incision can result in a thick or obvious scar. In breast cancers involving the outer aspect of the breast, a periareolar incision can lead to better cosmetic results compared to using a radial or transverse incision, as the tension along the wound caused by the weight of the breast decreases (Dixon et al. 1997). Moreover, a periareolar incision can be included in the mastectomy specimen as mastectomy is finally required.

Round-block technique belongs to the oncoplastic surgery. Although it is useful for obtaining surgical margins and better cosmetic outcome, larger incision is needed and more complications can occur. Hypertrophic scars are more common in young patients (Lim et al. 2017; Ogawa 2014). Breast-conserving surgery via periareolar incisions would be an appropriate technique for minimal operation scar and better cosmetic outcome for patients who are suitable for breast-conserving surgery. Round-block technique can be more suitable for patients who have indications for mastectomy, but do not want to receive mastectomy.

The benefits of a periareolar incision are similar between malignant and benign tumors. However, as the quality of life after breast cancer surgery in breast cancer patients is influenced by body image, nearly invisible scars and less obvious deformity are important aspects. Nipple-sparing mastectomy via a periareolar incision had better cosmetic results, although it also resulted in a high rate of partial or total NAC necrosis based on the surgical technique (Regolo et al. 2008). In BCS for cancers far from the NAC, vascular complications of the NAC rarely occurred because the retroareolar tissue is preserved. Hence, a periareolar incision should not be over half of the areola circumference, in order to minimize vascular impairment of the skin flap (Kong et al. 2016).

In our study, BCS using a periareolar incision was performed successfully in 3 patients who had multifocal lesions. The total diameter including the multifocal lesions was < 3.2 cm. Although the presence of multifocal breast cancer is a known contraindication for BCS, its oncological safety for multifocal breast cancer has been demonstrated (Lim et al. 2009). Therefore, the main cancer that is far from the NAC and suspected multifocal lesions on preoperative images can be removed together via a periareolar incision.

Among the various types of incisions, a periareolar incision gives better cosmetic results (Kong et al. 2016). A periareolar incision is usually made during lumpectomy for central lesions to optimize cosmesis and obtain adequate cancer exposure. The results of this current preliminary study showed that BCS via a periareolar incision is feasible and safe for cancers far from the NAC. However, the surgical technique and the physician’s experience are important. Moreover, patients undergoing this procedure should be selected after considering several conditions. There is no report on the upper limit of the distance from the NAC to the tumor in previous studies. When the cancer is far from the areola or is located at the peripheral end, pushing the breast lightly from the tumor side towards the NAC is helpful. The size of the areola is even more important for an adequate view during surgery. The maximum length of the periareolar skin incision that can be allowed is determined by the size of the areola, because it should not be over half of the total circumference. Moreover, it can help in determining the upper limit of the extent of tissue that can be excised in lumpectomy via a periareolar incision as the specimen is removed through the periareolar incision. After dissecting the subcutaneous fat below the main cancer, most cancers are palpable. However, if the cancer is too small to be palpated before surgery, preoperative localization of the tumor may be required; it is not routinely needed.

We successfully performed BCS for breast cancers far from the NAC in most patients; the cancers that were > 10 cm away from the NAC were included in this study. However, the small sample size is a limitation of this study. Even the retrospective nature of the study is a limitation, although further studies should be performed based on the positive results of this study. Moreover, although whole breast irradiation is usually performed after lumpectomy, the long-term local recurrence rate should be carefully observed and followed up. This technique was performed by a well-trained breast surgeon. Cosmetic results and complications may depend on the high experience of the surgeon.

## Conclusions

For cancers located far from the NAC, BCS via a periareolar incision is feasible and oncologically safe. Moreover, it also results in very good cosmetic outcomes in selective patients. The diameter of the lesions to be removed, the areola size, and the patient’s need for cosmesis should be considered. Long-term follow-up studies should evaluate whether that oncologic outcome of BCS via a periareolar incision for breast cancers far from the NAC is similar to that obtained with BCS using traditional incisions; the results will determine if BCS via a periareolar incision for breast cancers may be a better treatment option.

## Data Availability

The datasets during and/or analyzed during the current study are available from the corresponding author on reasonable request.
